# Depression research: where are we now?

**DOI:** 10.1186/1756-6606-3-8

**Published:** 2010-03-10

**Authors:** Saebom Lee, Jaehoon Jeong, Yongdo Kwak, Sang Ki Park

**Affiliations:** 1 Department of Life Science, Division of Molecular and Life Science, Pohang University of Science and Technology, Pohang, Republic of Korea

## Abstract

Extensive studies have led to a variety of hypotheses for the molecular basis of depression and related mood disorders, but a definite pathogenic mechanism has yet to be defined. The monoamine hypothesis, in conjunction with the efficacy of antidepressants targeting monoamine systems, has long been the central topic of depression research. While it is widely embraced that the initiation of antidepressant efficacy may involve acute changes in monoamine systems, apparently, the focus of current research is moving toward molecular mechanisms that underlie long-lasting downstream changes in the brain after chronic antidepressant treatment, thereby reaching for a detailed view of the pathophysiology of depression and related mood disorders. In this minireview, we briefly summarize major themes in current approaches to understanding mood disorders focusing on molecular views of depression and antidepressant action.

## Introduction

Mood disorders such as major depression and bipolar disorders are the most common psychiatric disorders in modern society. About 16% and 1% of the population are estimated to be affected by major depression and bipolar disorder one or more times during their life time, respectively [1]. The presence of the common symptoms of these disorders are collectively called 'depressive syndrome' and includes a long-lasting depressed mood, feelings of guilt, anxiety, and recurrent thoughts of death and suicide [2]. The genetic contribution to the manifestation of depression has been estimated as 40-50% [3]. However, combinations of multiple genetic factors may be involved in the development of depression, because a defect in a single gene usually fails to induce the expression of multifaceted symptoms of depression [4]. Also, various non-genetic factors such as stress, affective trauma, viral infection, and neurodevelopmental abnormalities increase the complexity of the pathogenesis of the disease. Thus, extensive studies have led to a variety of hypotheses for the molecular mechanism of depression, but a definite pathogenic mechanism has yet to be defined.

The 'monoamine hypothesis,' which suggests a deficiency or imbalances in the monoamine neurotransmitters, such as serotonin, dopamine and norepinephrine, as the cause of depression has been the central topic of depression research for approximately the last 50 years. This hypothesis has been initiated and supported by the fact that early versions of antidepressants including tricyclics and monoamine oxidase inhibitors have the common effect of acutely enhancing monoamine function [5-7]. Recent development of the selective serotonin reuptake inhibitors (SSRIs) as effective antidepressants has further strengthened the hypothesis [6,8]. However, unresolved complexity of the current antidepressants remains. First, antidepressants are effective in less than 50% of patients, and recently discovered drugs have failed to enlarge the extent of applicable patients [2]. Second, chronic treatment with antidepressants is required for clinical effects, and the reason for this is unknown [9]. Third, depression medications as well as mood stabilizers show a wide spectrum of undesired side effects.

In particular, because clinical effects of antidepressants that acutely modify monoamine systems are significantly delayed, it is now believed that an adaptation of downstream events, including lasting changes in gene expression by chronic treatment, underlie the antidepressant efficacy [10]. This phenomenon suggests that there is probably not a simple relationship between biogenic amines and depression postulated by classical monoamine hypothesis. The complexity may be due to multiple factors, which is likely because depression is a group of disorders with several underlying pathologies. Also, expression of depression symptoms may require disturbances in certain neurotransmitter systems that are functionally interconnected to each other at multiple levels. Taken together, while it still has to be emphasized that the initiation of antidepressant efficacy may be mediated by acute changes in monoamine systems, apparently, the focus of current research is moving toward molecular mechanisms that underlie long-lasting downstream changes in the brain after chronic antidepressant treatment, thereby reaching for a detailed view to the pathophysiology of depression and related mood disorders. In this minireview, we summarize major themes in current approaches to understanding depression and related mood disorders.

### Gene-environment interactions

As a way to discovering genes predisposing to depression, geneticists have long been searching for gene variants that play a role in the response to life stresses, a critical environmental factor for the onset of depression, which would be an example of 'gene-environment interaction': whereby an environmental factor is filtered through the activity of a gene to confer differential susceptibility to depression among individuals. To this end, polymorphisms in the serotonin transporter (5-hydroxyltryptamine transporter, 5-HTT) gene have been extensively analyzed. It has been reported that the expression level of 5-HTT from the 5-HTT gene is influenced by polymorphisms in the 5'-flanking region (5-HTT gene-linked polymorphic region, 5-HTTLPR) and in the variable number tandem repeat (VNTR) of the second intron [11,12]. In particular, a short variant of 5-HTTLPR appears to be associated with repressed transcriptional activity of the promoter, decreased 5-HTT expression, and decreased 5-HT uptake when compared with a long variant of 5-HTTLPR [13]. Significantly, genetic studies have shown that these polymorphisms are associated with major depressive disorder in human [14]. Moreover, a longitudinal study with 847 New Zealanders has shown that a short allele of 5-HTTLPR variants is associated with an increase in susceptibility to depression in response to life stresses such as job losses or divorces [15]. Strikingly, in this study, the polymorphism is influential only when the subjects are in significant life stresses, suggesting that 5-HTT may be a connecting point between individual's genetic makeup and environmental triggers of depression. These observations were further strengthened by study showing that increased depression scores in maltreated children without social supports are associated the short allele of 5HTTLPR [16].

However, the insight from these studies does not appear to be fully supported by other studies. The association of allelic variation in VNTR of 5-HTT gene with the susceptibility to depression was not consistently detected in some analyses [17,18]. A meta-analysis showed that polymorphisms in 5-HTTLPR and the second intron are actually found in depressed patients but the strength of association does not reach a statistical significance [19]. An extensive study using 1206 twins also failed to find a main effect of 5-HTTLPR, or an interaction between the 5-HTTLPR genotype and stressful life events on major depression [20]. Moreover, a recent meta-analysis using 14 comparable studies has yielded no evidence that the serotonin transporter genotype alone or in interaction with stressful life events is associated with an elevated risk of depression [21]. The mixed results from these studies reveal the potential weakness of the 'candidate gene' approach focusing on a specific gene variant to elucidate gene-environment interactions, and thus add importance on unbiased whole-genome scan approach, especially when a disease with polygenic nature, such as depression and related mood disorders, is concerned.

### Stress response circuits

Chronic stress is an important component in depression even though it does not seem to function as a necessary or sufficient factor. From this point of view, the hypothalamic-pituitary-adrenal (HPA) axis, a core neuroendocrine circuit for managing stress in the body, has been a topic of interest in depression research [22]. Corticotrophin-releasing factor (CRF) secreted from the paraventricular nucleus of the hypothalamus enhances secretion of adrenocorticotrophin (ACTH) from the pituitary [22,23], and subsequently, glucocorticoid is secreted from the adrenal cortex, impacting neurobehavioral functions of various brain regions [2]. The HPA axis forms a feedback loop via certain brain regions such as the hippocampus and amygdala [24]. It was reported that hypercortisolemia, a persistent upregulation of blood glucocorticoid levels, increases the excitotoxicity of CA3 pyramidal neurons in the hippocampus, resulting in dendritic atrophy, reduction in spinogenesis, apoptosis of neurons, and possibly inhibition of adult neurogenesis [25]. These functional abnormalities of hippocampal neurons caused by chronic stress can reduce the inhibitory tone on the HPA-axis, which results in hyperactivity of the HPA-axis [23]. Notably, hyperactivity of HPA-axis is evident in approximately half of depressed patients and chronic treatment with antidepressants often reverses this phenomenon [23,26]. Furthermore, evidence from animal studies suggests that chronic treatment with antidepressants appears to contribute to the recovery of the abnormal function of the hippocampus by increasing neurogenesis [27,28].

In this regard, one research direction is to evaluate the therapeutic potentials of weakening of the functions of the HPA axis. The obvious targets are CRF receptors expressed in the pituitary and glucocorticoid receptors expressed in the hippocampus and other brain regions, because those receptors are core components in the HPA axis and the associated feedback loop [24,29-32]. In a similar context, vasopressin receptors have also emerged as alternative targets [33,34]. Vasopressin is a neuropeptide that enhances CRF function and works through vasopressin receptors expressed in the amygdala and other parts of the limbic system. Also, a single nucleotide polymorphism (SNP) of vasopressin 1b (V1b) receptor has protective effects against major depressive disorder [35]. Intriguingly, antagonism of CRF receptors, glucocorticoid receptors, and vasopressin receptors appear to exhibit antidepressant effects in experimental animals. The applicability to human patients remains to be further refined.

### Neurotrophic factors

Long-term stress appears to reduce the expression level of brain derived neurotrophic factor (BDNF) in the hippocampus [36]. Also, in a post-mortem study of depressed patients, a reduction in BDNF expression was reported [37]. In addition, polymorphisms of BDNF gene are associated with neuroticism, a personality trait linked to increased susceptibility to depression [38]. A family-based association study showed that polymorphisms in BDNF genes are related to bipolar disorders [39]. Conversely, a chronic treatment with antidepressants not only enhances the BDNF level but also increases the stress resistance in animals [40,41]. These observations provided a basis for 'neurotrophism theory' stating that depression is caused by a deficit in neurotrophic factors, and antidepressants neutralize this deficit. This theory may be intimately related to neuronal damages in the hippocampal region caused by hyperactivity of stress response circuits aforementioned. Because BDNF is known to enhance synaptic plasticity in various brain regions [42,43], it is reasonable to postulate that improving BDNF function may be beneficial to the hippocampal neurons that are susceptible to stress-induced damages. Supporting this idea, direct injection of BDNF into the hippocampus of experimental animals induces behavioral changes similar to antidepressant treatment [41]. Thus, BDNF and its receptor TrkB, have become promising targets of novel-type anti-depression therapies.

Despite these observations, a possible causative relationship between BDNF function and the pathogenesis of depression or antidepressant efficacy requires further clarification. For example, while the antidepressant efficacy is suppressed in experiments using inducible BDNF knock-out mice, depression-related behaviors are only seen in females, showing significant gender differences [36]. Moreover, forebrain-specific conditional TrkB receptor knockout mice do not exhibit depression-related behaviors such as increased behavioral despair in the forced swim test [44], whereas it has been demonstrated that activation of TrkB receptor is required for antidepressant-induced behavioral effects [45]. Thus, the relationship between the loss of BDNF activity and the expression of depressive symptoms is not in a simple correlation. Nevertheless, the potential value of the neurotrophic theory as a basis for the design of new form of anti-depression therapies cannot be excluded by the complexity of the current experimental results.

### Histone modifications

One poorly understood characteristic of antidepressants is the long delay before the onset of positive effects in patients [10]. This phenomenon is often attributed to the slow development of adaptation in the relevant neurons that underlies the beneficial effect of the drugs. The identity of the adaptation is not clear yet, but enduring changes in the state of chromatin are thought to be involved. Chronic electro-convulsive shocks that are effective for some depressed patients also induce changes in wide range of the histone modification patterns in experimental animals [46]. One locus with prominent changes is BDNF, and in conjunction with the suggestion of BDNF as a potential target for design of new antidepressants, the epigenetic control of BDNF expression has been extensively analyzed in the context of the expression of depression and chronic antidepressant treatments. In the rat hippocampus, chronic electro-convulsive shocks increase acetylated histone H3 at the BDNF promoters 3 and 4, and these modifications appear to be correlated with increased expression of BDNF and CREB [46]. This upregulation has been linked to the effects of antidepressants in animal studies [28,47]. Moreover, chronic defeat stress, an experimental model for depression, elicits selective downregulation of some BDNF splice variants, in the hippocampus [28]. This downregulation appears to be due to induction of H3-K27 dimethylation, a histone code for transcriptional repression [28,48]. Conversely, an antidepressant treatment reverses repression of BDNF expression likely by inducing H3 acetylation and H3-K4 methylation, acting as histone codes for transcriptional activation, at the BDNF promoter region [49]. During this whole process, roles for histone deacetylases (HDACs) seem to be crucial because chronic antidepressant treatment downregulates HDAC5, and overexpression of HDAC5 in the hippocampus prevents its antidepressant effect [28].

HDAC inhibitors have thus received attention for their potentials as promising therapeutics for depression and related mood disorders. HDAC inhibitors are members of four families: the short chain fatty acids (e.g. sodium butyrate (SB), phenylbutyrate, and valproic acid (VPA)), the hyroxamic acids (e.g. TSA and suberoylanilide hydroxamic acid (SAHA)), the epoxyketones (e.g. trapoxin), and the benzamides. One of the most widely used mood stabilizers is VPA. As VPA is known to have an inhibitory activity on HDAC1 and presumably other HDACs [50], it has been proposed that its mood stabilizing efficacy may be mediated at least in part by histone modifications. Another study showed that HDAC inhibitors such as VPA, SB, and TSA increase BDNF expression in the brain [51]. Thus, epigenetic mechanisms, especially histone modification, seem to have the potential to provide new mechanistic insights into the expression of depression and novel treatments for depression and related mood disorders.

### Adult hippocampal neurogenesis

Brain imaging studies showing reduced hippocampal volume in depressed patients have provided a platform for investigating adult neurogenesis in the context of the pathogenesis of depression [52]. The hypothesis states that chronic stresses and other depression-inducing stimuli decrease neurogenesis [53-55], whereas antidepressant efficacy may rely on an increase in neurogenesis [54-56]. Adult neurogenesis is restricted to the subventricular zone and subgranular zone of the hippocampus [57], and this emphasizes the potential importance of hippocampal neurogenesis during the onset as well as during the treatment of depression. Supporting this idea, various animal models of depression, such as learned helplessness, chronic mild stress, and psychosocial stress, are associated with reductions in hippocampal neurogenesis [58-60]. Conversely, chronic antidepressant treatment not only increases neurogenesis but also supports survival of newborn neurons [61]. It has also been shown that the antidepressant efficacy of tricyclics, imipramine, and SSRIs requires hippocampal neurogenesis in rodents [58,62,63]. Furthermore, chronic fluoxetine treatment appears to increase the number of synapses in the pyramidal cell layers and block the decrease in spine density in the dentate gyrus and other hippocampal cell layers [64]. Notably, enriched environments, which is known to enhance hippocampal neurogenesis [65], decrease depression-related behaviors in rodents [66,67].

The expression level of BDNF deserves attention when examining the molecular mechanisms underlying the antidepressant-mediated increase in neurogenesis. As described above, in various animal models of depression, the BDNF level is decreased [40], whereas chronic antidepressant medication and electro-convulsive shocks increase the levels in the hippocampus [28,46]. A recent study showed that CREB, a transcription factor that regulates expression of CRE-containing target genes including BDNF, is also upregulated and activated in hippocampus by chronic antidepressant treatment [2,53,68,69]. However, the cause and effect relationship among the induction of CREB and BDNF, the neurogenesis, and behavioral effects of antidepressants remains to be further investigated.

Recent studies demonstrated that long-term administration of mood stabilizers such as lithium, valproic acid, and carbamazepine also enhances adult hippocampal neurogenesis [70-72]. Lithium directly inhibits glycogen synthase kinase-3 (GSK-3) and inositol signaling [73]. VPA enhances gene expression likely by inhibiting HDACs, indirectly blocks GSK-3 activity, and suppresses inositol signaling [71,74-76]. Although it remains unclear whether the GSK-3 and inositol signaling are actually linked with clinical effects of mood stabilizers, the data suggest a common molecular pathway constituting the pathophysiology of depression and related mood disorders that converges on adult hippocampal neurogenesis.

### Substance withdrawal

Various drugs such as alcohol, psychostimulants, opiates and *N*-methyl-D-aspartate (NMDA) receptor antagonists generate a physiological response called withdrawal symptoms during abstinence in humans and experimental animals [77-80]. The characteristics of affective symptoms caused by drug withdrawal and major depressive disorder are strikingly similar [80]. Depressed mood and anhedonia are commonly present with both drug abstinence and depressive disorders [81]. Hyperphagia, hypersomnia, feelings of fatigue, and suicidal ideation are also observed in both conditions [82,83]. Disruptions of the HPA axis are also seen during drug withdrawal, and are accompanied by increased levels of cortisol and elevated cerebrospinal levels of CRF [84]. In addition, elevated levels of cortisol, ACTH and β-endorphin during early cocaine withdrawal resemble those in depressed patients [85]. Brain-imaging studies using positron emission tomography (PET) and functional magnetic resonance imaging (fMRI) have revealed that methamphetamine withdrawal induces decreased glucose metabolism in the anterior cingulate cortex and insula, and increased metabolic activity in the amygdala and orbitofrontal cortex, all of which are frequently observed in clinical depression [86].

Much evidence shows that depression and related mood disorders are accompanied by abnormalities in dopaminergic transmission in the nucleus accumbens (NAc) and ventral tegmental area (VTA), regions that are core parts of the brain reward circuit [87]. It is well established that depressed patients have difficulties in the expression of pleasure and acquisition of motivation, which are mainly governed by a normal NAc-VTA dopamine circuit [88]. Consistently, it has been shown that a deregulation of dopamine D2 receptor signaling results in depression-like behaviors in experimental animals [89], and that neuronal nitric oxide synthase (nNOS) knockout mice with altered dopamine D1 receptor signaling exhibit decreased depression-related behaviors [90]. Because nearly all drugs of abuse directly or indirectly activate monoaminergic neurotransmission in the limbic system, resulting in reward sensations [91,92], it has been postulated that counter-adaptations may occur in opposition to the reward effects with chronic drug intake, generating cognitive, motivational, and affective impairments, including depression-like symptoms during the drug withdrawal period [93].

As described above, in many ways, depressive mood subsequent to drug withdrawal shares common characteristics, such as neuro-hormonal changes, regional brain activity, and pharmacological responses, with clinical depression. However, it needs to be emphasized that the onset, course, duration, and other factors such as involvement of substances diagnostically distinguishes substance-induced mood disorders from major depressive disorders [94,95]. Some experimental data also hint at differences between these conditions at the molecular level, demanding cautions when interpreting the related observations. For example, dopamine transporter densities are increased in the striatum in both cases [96], but serotonin transporter densities are elevated in the brainstem during the early stage of cocaine abstinence [97], but not in clinical depression [98]. Also, some abstinent drug addicts have been treated with antidepressant drugs to reduce drug craving, but the positive effect of these drugs needs further validation [99]. Nonetheless, insights from these views not only tell us that brain reward circuits composed of the mesolimbic system are potentially important in understanding depression, but also provide a useful behavioral readout for depressive mood in experimental animals.

### Circadian rhythms

Circadian rhythm is a roughly 24-hour cycle of biochemical, physiological, and behavioral processes under control of internal clock [100-102]. From the clinical point of view, a potential link between circadian rhythms and depression or related mood disorders has long been postulated. For example, it is relatively well known that insufficient length of light phase to entrain the circadian rhythm can be causative for the development of seasonal affective disorders [103,104]. Also, abnormal regulation of sleep/wake cycles, body temperature, blood pressure, and various endocrine functions under the control of circadian clock are prominent symptoms of mood disorders [102,105-110]. However, molecular mechanisms underlying the link are still largely unknown.

Recently, interesting observations have been made in the mutant mouse that has a deletion of 19th exon of *Clock *gene, a core component of molecular clock. The mouse exhibits hyperactive VTA dopaminergic neurons and behavioral phenotypes that are reminiscence of mania seen in bipolar disorder patients [111,112]. Moreover, lithium, a mood stabilizer for bipolar depression patients, effectively inhibits GSK3β, a core regulatory component in the molecular clock. Lithium also has an effect on the nuclear entry of Period-Cryptochrome heterodimers, a key process to form a negative loop in the molecular clock, likely through an inhibition of GSK3β activity. Furthermore, lithium appears to regulate activity of Rev-erb α that links the negative loop to the positive loop in the biological clock [113-116].

Potential links between circadian rhythm and the monoamine system are also reported. The synthesis and/or secretion of monoamine neurotransmitters and the function of their receptors are under influence of circadian rhythms. The circadian rhythmicity of dopamine transporter and tyrosine hydroxylase expression in dopaminergic neurons is also disrupted when the suprachiasmatic nucleus of the hypothalamus, the central part of endogenous clock, is damaged [117]. Moreover, monoamine oxidase-A (MAO-A) expression is regulated by dimer formation of Clock and Bmal1, and MAO-A activity accordingly shows a circadian rhythmicity [118]. Conversely, the expression of circadian genes such as *Clock, Per1*, and *Bmal1 *is stimulated when dopamine D1 receptor is activated, and suppressed when dopamine D2 receptor is activated in the limbic area [119]. Collectively, the molecular clock appears to be tightly interconnected with monoamine systems, which might explain symptomatic correlation between circadian rhythm and depression at the molecular level.

Although the relationship among the daily variations of mood, endogenous molecular clock, and the expression of depressive symptoms is complicated, normalization of the biological rhythms of a depressive individual could have a beneficial effect. In this regard, the recent development of agomelatine as an antidepressant is of great interest. Agomelatine is a potent agonist for melatonin receptors and has capacity to reset the internal circadian clock [120,121]. Intriguingly, it also exhibits antagonistic activity on 5-HT_2*C *_receptor, thereby indirectly enhancing the dopamine and norepinephrine neurotransmission [122-124]. Moreover, agomelatine affects differentially various stages of neurogenesis in the dorsal and ventral hippocampus [125]. Further understanding of the molecular basis of agomelatine action and its efficacy may provide interesting insight into the interface between circadian rhythm and pathophysiology of depression.

### Functional anatomy

Information on brain regions and neural circuitry responsible for the expression and progression of a disease is an important platform to better diagnose the disease and to properly interpret the observations obtained from molecular, cellular, and tissue experiments in the clinically relevant context. While various brain regions are known to be involved in regulation of mood or emotion, definite information on central neural circuits responsible for mood disorders is still incomplete, mainly because anatomical lesions in patients have been less consistently found relative to other various neurological disorders such as some neurodegenerative diseases. However, there are neuropathological and neuroradiological studies that have established interesting associations between mood disorders and structural abnormalities in the brain. For example, glial reduction was observed in anterior cingulate gyrus and neuronal abnormalities were detected in the dorsolateral prefrontal cortex in post-mortem neuropathological studies of mood disorder patients [126,127]. Radiological studies using MRI also revealed reduced volumes of orbitofrontal and subgenual anterior cingulate cortex [128-130], electrical stimulation of which correlatively elicits an antidepressant effect [131]. Most notably, reductions in hippocampal volume in depressed elderly patients were reported [132,133].

Recent brain imaging studies mainly using fMRI are adding information on brain regions that play important roles in depressive symptoms at the functional level [134]. Functional changes in brain regions such as prefrontal/cingulate cortex, hippocampus, striatum, amygdala, and thalamus are correlated with depression [52]. The neocortex and hippocampus also appear to play critical roles in the symptoms related to the cognitive deficits that are prevalent in depressed patients [55], and the nucleus accumbens and amygdala seem to be core regions for anhedonia and emotional memory-related symptoms [135,136]. The functional changes in the hypothalamus are also linked to sleep- and appetite-associated symptoms [137]. Research on these topics is now being accelerated by fast advances in brain imaging technologies, and the outcome, in combination with the information from the conventional anatomical studies, is driving the generation of a higher-resolution picture of the neural circuitry relevant to depression.

## Conclusion

**Figure 1:**
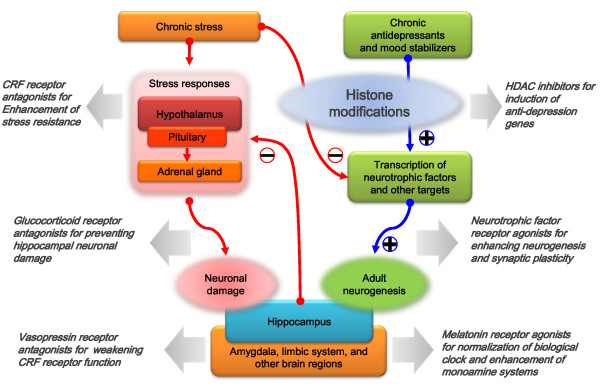
**Approaches to the development of antidepressants targeting non-monoaminergic components**. Chronic stress can cause hypercortisolemia which results in neuronal damages in the hippocampus, thereby weakening the feedback inhibition on HPA axis. Chronic stress also can inhibit the expression of neurotrophic factors through epigenetic mechanisms. On the other hand, chronic treatment of antidepressants and mood stabilizers can establish epigenomic environments that favor the expression of anti-depression genes. The targets may include genes for neurotrophic factors which prevent neuronal damages and enhance hippocampal neurogenesis. Some of approaches to the development of antidepressants targeting non-monoaminergic components are also shown.

A prerequisite for effective control of depression and related mood disorders is to understand their detailed molecular pathways. Although the classical stress model of depression and current understanding of antidepressant action appears to be partially linked via epigenetic mechanisms and hippocampal neurogenesis (Figure 1), obviously, the current picture of the pathophysiology of depression is largely incomplete, and thus many potential hypotheses are being generated and tested, forming fragmented neurobiological views of depression and related mood disorders. One major task in the field must be to integrate the relevant hypotheses to formulate a bigger picture of the pathophysiology of depression and related disorders. A key step may be to define the high-resolution neural circuitry of depression, which will provide a platform to better interpret the observations obtained from molecular, cellular, and tissue experiments at the organism level. Another critical step will be to identify 'depression genes' that are causative for depression. This will help us generate genetic animal models that may not only be critical for clarifying many issues in depression research using experimental animals, but may also be useful for assessing the potential efficacy of candidate antidepressants. Finally, the most challenging task in the field is to overcome the limitations of current therapies, which are only effective in a fraction of patients. It has long been expected that novel antidepressants targeting non-monoamine systems would enlarge the extent of treatable patients (Figure 1), but the progress still falls short of expectations, thereby leaving it as a pressing task in the field.

## Competing interests

The authors declare that they have no competing interests.

## Authors' contributions

SL, JJ, and YK collected information and participated in drafting the manuscript. SKP wrote the manuscript and coordinated the drafting process. All authors read and approved the final form of the manuscript.
